# Fluid and structure coupling analysis of the interaction between aqueous humor and iris

**DOI:** 10.1186/s12938-016-0261-3

**Published:** 2016-12-28

**Authors:** Wenjia Wang, Xiuqing Qian, Hongfang Song, Mindi Zhang, Zhicheng Liu

**Affiliations:** 1 School of Biomedical Engineering, Capital Medical University, Beijing, China; 2 Beijing Key Laboratory of Fundamental Research on Biomechanics in Clinical Application, Capital Medical University, Beijing, China; 3 School of Mechanical and Vehicular Engineering, Beijing Institute of Technology, Beijing, China

**Keywords:** Aqueous humor, Iris deformation, Intraocular pressure, Mechanical properties, Fluid–structure interaction

## Abstract

**Background:**

Glaucoma is the primary cause of irreversible blindness worldwide associated with high intraocular pressure (IOP). Elevated intraocular pressure will affect the normal aqueous humor outflow, resulting in deformation of iris. However, the deformation ability of iris is closely related to its material properties. Meanwhile, the passive deformation of the iris aggravates the pupillary block and angle closure. The nature of the interaction mechanism of iris deformation and aqueous humor fluid flow has not been fully understood and has been somewhat a controversial issue. The purpose here was to study the effect of IOP, localization, and temperature on the flow of the aqueous humor and the deformation of iris interacted by aqueous humor fluid flow.

**Methods:**

Based on mechanisms of aqueous physiology and fluid dynamics, 3D model of anterior chamber (AC) was constructed with the human anatomical parameters as a reference. A 3D idealized standard geometry of anterior segment of human eye was performed. Enlarge the size of the idealization geometry model 5 times to create a simulation device by using 3D printing technology. In this paper, particle image velocimetry technology is applied to measure the characteristic of fluid outflow in different inlet velocity based on the device. Numerically calculations were made by using ANSYS 14.0 Finite Element Analysis. Compare of the velocity distributions to confirm the validity of the model. The fluid structure interaction (FSI) analysis was carried out in the valid geometry model to study the aqueous flow and iris change.

**Results:**

In this paper, the validity of the model is verified through computation and comparison. The results indicated that changes of gravity direction of model significantly affected the fluid dynamics parameters and the temperature distribution in anterior chamber. Increased pressure and the vertical position increase the velocity of the aqueous humor fluid flow, with the value increased of 0.015 and 0.035 mm/s. The results act on the iris showed that, gravity direction from horizontal to vertical decrease the equivalent stress in the normal IOP model, while almost invariably in the high IOP model. With the increased of the iris elasticity modulus, the equivalent strain and the total deformation of iris is decreased. The maximal value of equivalent strain of iris in high IOP model is higher than that of in normal IOP model. The maximum deformation of iris is lower in the high IOP model than in the normal IOP model.

**Conclusion:**

The valid model of idealization geometry of human eye could be helpful to study the relationship between localization, iris deformation and IOP. So far the FSI analysis was carried out in that idealization geometry model of anterior segment to study aqueous flow and iris change.

## Background

Glaucoma is the primary cause of incurable blindness in the world [1]. It generally develops from a resistance of aqueous humor outflow, leading to an increase in IOP [2]. The retina ganglion cells progressively damages, as a consequence of this, visual field reduced and finally grows blind [3]. In primary angle-closure glaucoma (PACG), the iris is abnormally positioned and physically impedes aqueous humor outflow through the trabecular meshwork. PACG derives its name from the narrow anterior chamber angel, defined by the iris anterior surface and the posterior surface of the corneoscleral shell.

**Fig. 1:**
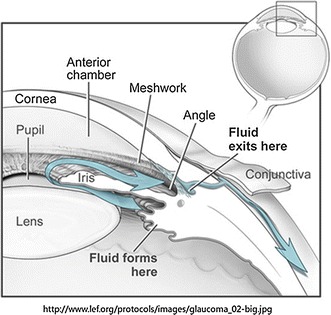
The main outflow pathway of aqueous humor from generation to outflow. The aqueous humor is actively secreted by the ciliary body, and it processes into the posterior chamber, passes through the pupil, enters the anterior chamber, and eventually exits, primarily through the trabecular meshwork

Aqueous humor is a kind of transparent fluid, which circulates through the anterior segment and provides nutrition for avascular ocular tissues, such as lens, anterior vitreous, cornea and trabecular meshwork (Fig. 1). Aqueous humor is actively secreted at a rate of 1.5–3.0 µL/min by the ciliary, and then enters into the posterior chamber, moves through the pupil, processes into the anterior chamber, and eventually flows out, primarily exits the trabecular meshwork, which residence in a time of about 100 min [4, 5].

The interest in comprehensive the iris mechanics develops from the influence of abnormal iris morphology on specific ocular disorders. For instance, in angle closure, the iris bows anteriorly, and the passively deformed shape and position of the iris restrict aqueous humor outflow, leading to a high intraocular pressure [6]. A recent study suggested that biomechanical properties of the iris may play an important role in the development of acute angle closure attacks [7]. Therefore, it is necessary to understand the changes of the mechanical properties of iris in the course of glaucoma. However, the fluid dynamics of aqueous humor in the anterior chamber and the change of the iris passive deformation have not been fully understood. Consequently, numerical simulation of fluid structure interaction in anterior chamber could present enlightening information on ocular diseases such as glaucoma where the outflow of AH is obstructed.

Previous report carried out several simulations and mathematical models to research the mechanism of aqueous humor fluid flow in anterior chamber, such as the distribution of temperature and velocity [8–13]. Heys et al. [14] created a numerical model of anterior segment to research on the mechanical mechanism interaction between aqueous humor and iris. Considering the miosis and blinking, etc., they analyzed the aqueous flow in anterior chamber. Repetto et al. [15] used a numerical model to study aqueous flow in anterior chamber whether the presence of a phakic intraocular lenses. However, the lack of verification to the validity and reliability of the finite-element analysis models might limit their accuracy. In addition, none of these simulations used fluid–structure coupling method to analysis the relationship between iris deformation and IOP in human eyes.

The goal of this research was to develop a 3D eye model based on idealization geometry of human eye and to establish the characteristics of aqueous humor flow under physiological and pathological conditions such as primary angle-closure glaucoma. This device will make a foundation for further simulations and evaluations of different influence factors of glaucoma.

## Methods

### Geometry description

**Fig. 2:**
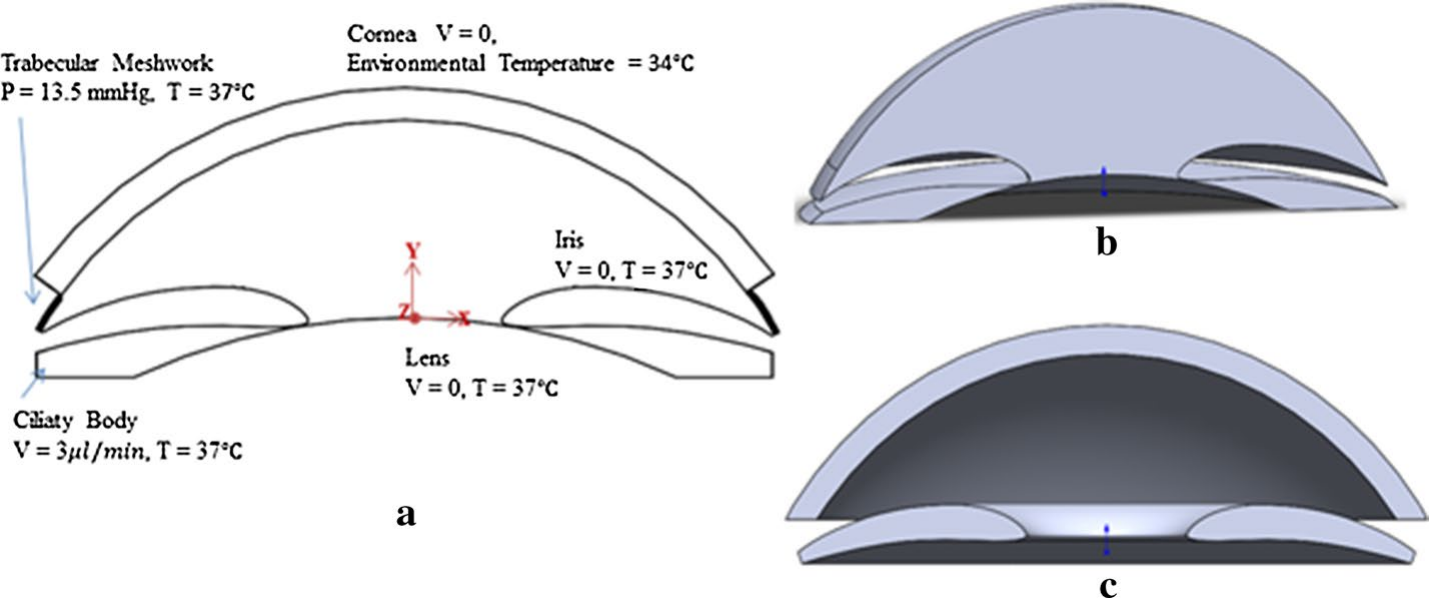
**a** A two-dimensional representation of the idealization model and boundary conditions. **b** Three-dimensional representation of the aqueous humor model domain based on **a** and created by software SolidWorks2014, and the corresponding structure model (cornea and iris) is shown in **c**. In **a** *P* is pressure, *V* is stream velocity, *T* is temperature. *V = 0* means no-slip boundary condition on cornea surface, lens surface and iris root

The model of the anterior segment studied here is similar to that of Repetto et al. [15]. The geometry model was created by using software SolidWorks2014 shown in Fig. 2. In the Fig. 2a, we present a two-dimensional scheme of idealization geometry of human eye. Specifically, we assume that the anterior segment is axisymmetric, bounded by the cornea, iris, lens, and trabecular meshwork. The posterior surface of cornea and anterior part of the lens are regarded as two segments of the spheroid with a radius of 6.8 and 10 mm, such that their centers are 3 mm apart, and the cornea thickness is 0.5 mm [16]. Most of the scales of iris are based on the ultrasound biomicroscopy measurements by Wang et al. [17], which maximum height is about 0.4 mm. The additional geometry size and corresponding parameter values used for the finite element analysis (FEA) calculations are given in Table 1.

**Table 1:** Parameter values applied in the simulations

Quantity	Value	References
Aqueous properties		
Density *ρ*	1000 kg/m^3^	Water properties
Volumetric flux *Q*	3 µl/min = 5 · 10^−8^ kg s^−1^	[28, 29]
Dynamic viscosity *v*	0.001 kg m^−1^ K^−1^	[30]
Specific heat *Cp*	4.178 · 10^3^ J kg^−1^ K^−1^	[31]
Thermal expansion coefficient *α*	0.0003 K^−1^	
Thermal conductivity *k*	0.578 W m^−1^ K^−1^	[32]
Viscosity *µ*	0.001 kg^−1^ s^−1^	[30]
Geometric characteristics of the domain		
Diameter of the anterior chamber D_C_	13 mm	[15]
Maximum height of chamber h_C_	2.63 mm	
Minimum radius of curvature of the posterior cornea R_C_	6.8 mm	
Radius of curvature of the natural lens R_L_	10 mm	
Height of the iris–lens channel	0.1 mm	
Angle between cornea and iris	30°	
Structure properties		
Young’s modulus of cornea	19.1 MPa	[33, 34]
Poisson’s ratio of cornea	0.49	[35]
Density of cornea	1143 kg/m3	[36]
Young’s modulus of iris	0.027 MPa	[37]
Poisson’s ratio of iris	0.49	

### Simulation device production

**Fig. 3:**
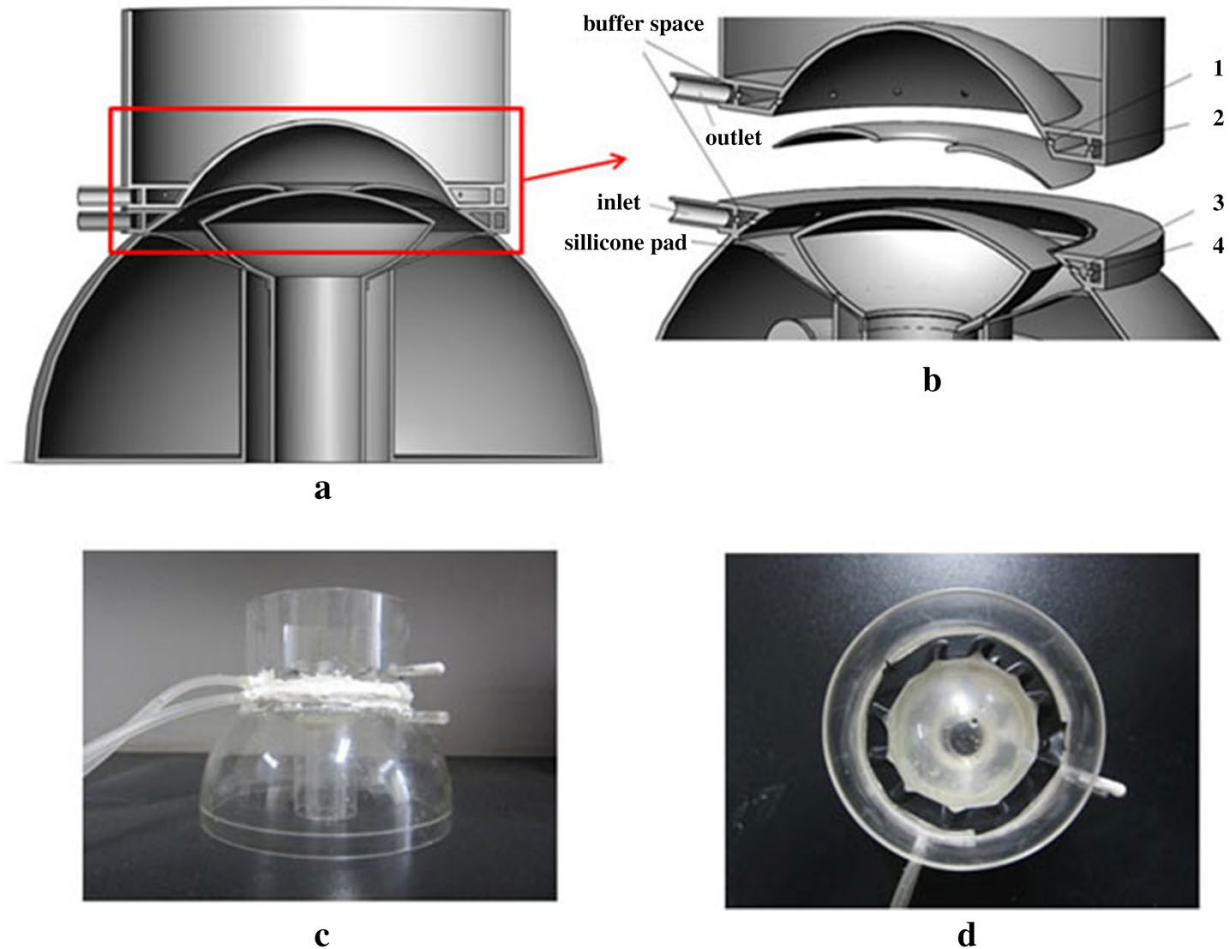
**a** A three-dimensional model of simulation device manufacted by 3D printing based on the idealization geometry in Fig. 2, magnified in 5 times. **b** The device model designed an inlet pipe, an outlet pipe, a silicone pad, two buffer spaces, a sink and many small holes (*1*, *2*, *3*, and *4*). The fluid is infused by the inlet pipe into the inlet buffer space, through 2 circle of small holes (*3*, *4*) evenly into the posterior chamber from all directions, passes through the pupil, enters the anterior chamber, uniform inflow of small holes (*1*, *2*) and outlet buffer space, and eventually exits through the outlet pipe. The silicone pad can prevent water leakage. And the function of the water sink is to avoid the refraction of the laser. The device produced by 3D printing is shown in **c** and **d**

PIV technology and 3D printing, as the new technology, are increasingly applied to the biomedical field, and helping to create a model for effectively experiments that cannot be achieved in animals [18, 19]. In order to verify the validity of the finite element model, we designed a simulation device based on the idealization geometry of human eye magnified in 5 times. Figure 3a shows a 3D view of the inner face of simulating equipment model. The amplification of the aqueous flow area in anterior segment is shown in Fig. 3b. The device (Fig. 3c, d) was produced using 3D printing technology, which is provided by Beijing innovo Technology Co. Ltd.

Compared with the idealization geometry, a cylindrical water sink with a 78 mm diameter was created outside the cornea of the simulation device to reduce the laser reflection on cornea surface. We also designed an inlet and an outlet channel to simulate the inflow and the outflow of aqueous humor. In order to make the fluid flow balance and uniform through the simulation device, outside of the inlet buffer space and the outlet buffer space are designed with many small holes. A column below the lens plays a supporting role, engendering a 10 µm gap between lens and iris. A silicon wafer, which to prevent the downward leakage of fluid flow, is provided substantially entirely on the back surface of the lens, connected under the holes of inlet.

### PIV measurement

The simulation device of anterior segment is used to simulate the physiological environment of aqueous humor fluid flow. The flow field in anterior chamber is measured by particle image velocimetry technology. The pivotal device in our study is image velocimetry system, provided by Beijing Institute of Technology, which can be found in Yang et al. [20].

**Fig. 4:**
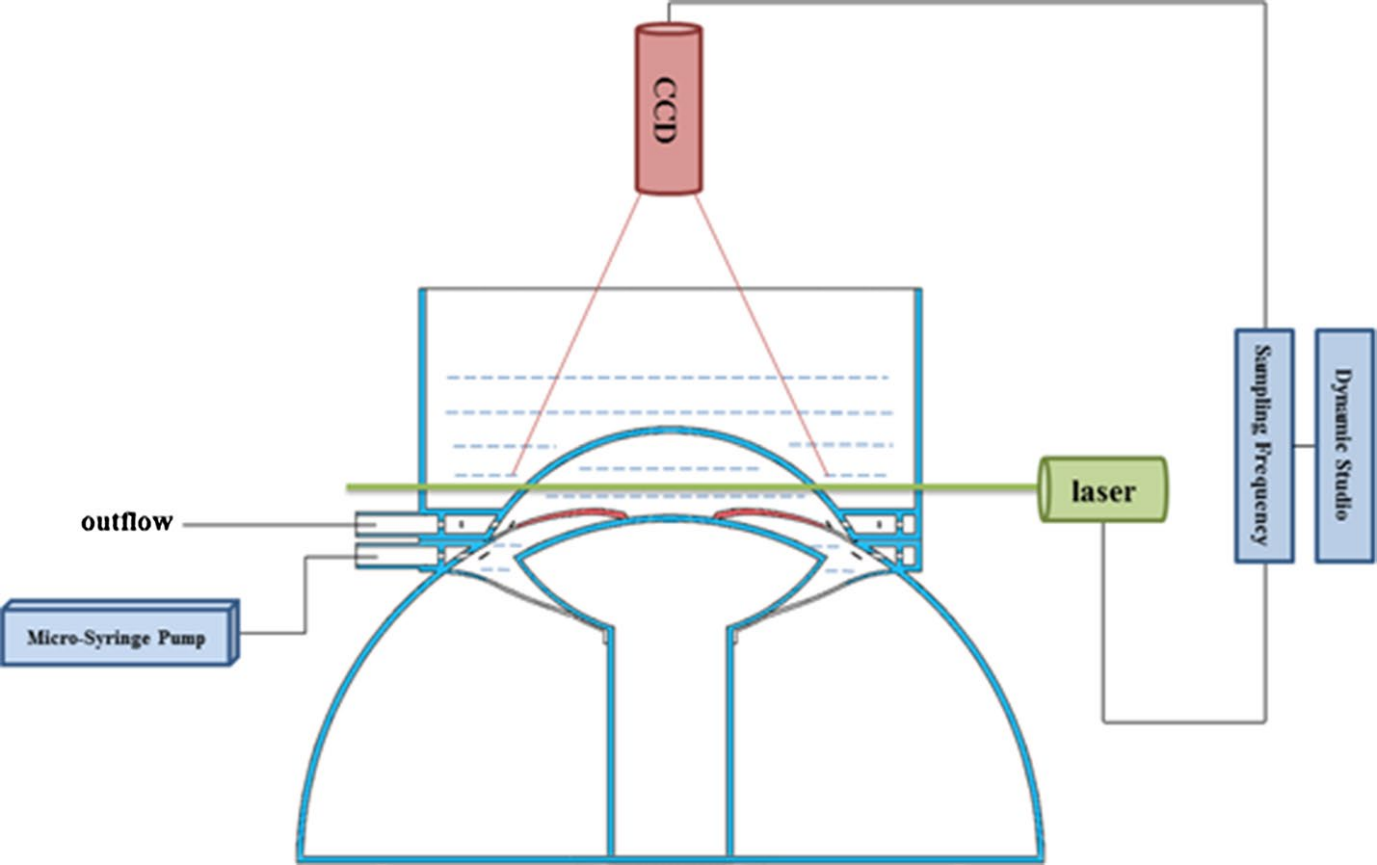
Connection diagram of experimental apparatus

Polystyrene particle is selected as the tracer particle, easily passing through the pupil with 10 microns in diameter. They can be isotropic spread and show a real following feature in intraocular fluid [21]. Devices connected are as shown in Fig. 4. Polystyrene particles solution was uniformly extracted with the medical syringe (20 ml) injected to the device by using the micro-injection pump at different speeds (5000, 10,000, 15,000, 20,000, 25,000, 30,000, 35,000 µl/min). The flow was recorded and processed by PIV equipment.

Compared with the velocity calculated by ANSYS Fluent14.0, utilized the 5 times geometry model, to verify the validity of the finite element model.

### Governing equation

The motion of aqueous humor in anterior segment is treated as a steady and incompressible Newtonian viscous fluid in this study, governed by the equations of momentum (Navier–Stokes) and continuity.

1a $$\rho \left( {v \cdot \nabla v} \right) = - \nabla p + \mu \nabla^{2} v + \rho g,$$

1b $$\nabla \cdot u = 0,$$

where v stands for the velocity vector, *p* represent pressure, *ρ* is fluid density, *μ* is fluid dynamic viscosity, and g is gravitational acceleration, with the magnitude g = |g|.

In the case of buoyancy-driven flow, the equations above are coupled to the energy equation

2 $$\frac{\partial T}{\partial t} + \left( {u \cdot \nabla } \right)T - \frac{k}{{\rho C_{p} }}\nabla^{2} T = 0,$$

where *T* stands for temperature, *k* represents the fluid thermal conductivity, and *C*_*p*_ defines the specific heat at constant pressure. We have neglected the heat production due to mechanical energy dissipation in Eq. 2, which is negligible in the present condition.

The Boussinesq approximation was used to stand for the unfluence of buoyancy, because of the temperature variations. The approximation states that the density of fluid changes with temperature and has little correlation in pressure. The Boussinesq approximation was given by:

3 $$\rho = \rho_{0} \left[ {1 - \alpha \left( {T - T_{0} } \right)} \right],$$

where *ρ*_0_ stand for the density of fluid at a reference temperature $T_{0}$, and *α* defines the fluid volume expansion coefficient. Values are summarized in Table 1.

### Boundary condition

The cornea limbus, iris root, and lens are modeled as stationary boundaries, and impased no-slip boundary condition on the surfaces. The front and back surface of iris and the corneal endothelium are set as FSI surfaces. Set the plane XY to a plane of symmetry. The temperature at the cornea is assumed along the outer surface, generally considered to lie between 33 and 35 °C [22], and was defined to be 34 °C in this study. The buoyant force mechanism provided by temperature difference between iris (37 °C) and cornea drives aqueous humor in the anterior chamber.

Aqueous humor is simulated inflows from the ciliary body area at a rate of 10 e−8 kg/s (3 µl/min). The working IOP condition was defined by an IOP of 13.5 mmHg to represent the healthy model while the glaucomatous case was set of 27 mmHg of IOP. Thus, the static pressure forced at the trabecular meshwork was estimated 13.5 mmHg and 27 mmHg to simulate the healthy and the glaucomatous models. In our study, aqueous humor secreted from the ciliary body and the trabecular meshwork was assumed to be at 37 °C. The boundary conditions are presented in Fig. 2a. The model consisted of two elastic isotropic segments—iris and cornea, structure properties are given in Table 1.

A volumetric mesh was developed by ICEM CFD software (ANSYS Inc., Canomsburg, PA, USA) and exported to ANSYS Fluent (ANSYS Inc., Canonsburg, PA, USA) for the fluid simulation. The 3D model of fluid structure and solid structures (iris and cornea) are depicted in Fig. 2b, c. A group of 20 s was performed to simulate the process with a time step of 0.5 s. There is no need to set a too small time step, because the aqueous humor flow in human eye is relatively low. Since the time step of 0.5 s was small enough, it allowed us to have a better assessment of the pattern of stress and deformations at iris and cornea of the simulation. The distributions of stress, velocity and temperature in each region when the velocity inlet at a speed of 10e−8 kg/s and the IOP of 13.5 or 27 mmHg were modeled and compared by the post-processing software(ANSYS Inc., Canonsburg, PA, USA).

## Results

### Flow field induced by different velocity of inlet in simulation device

**Fig. 5:**
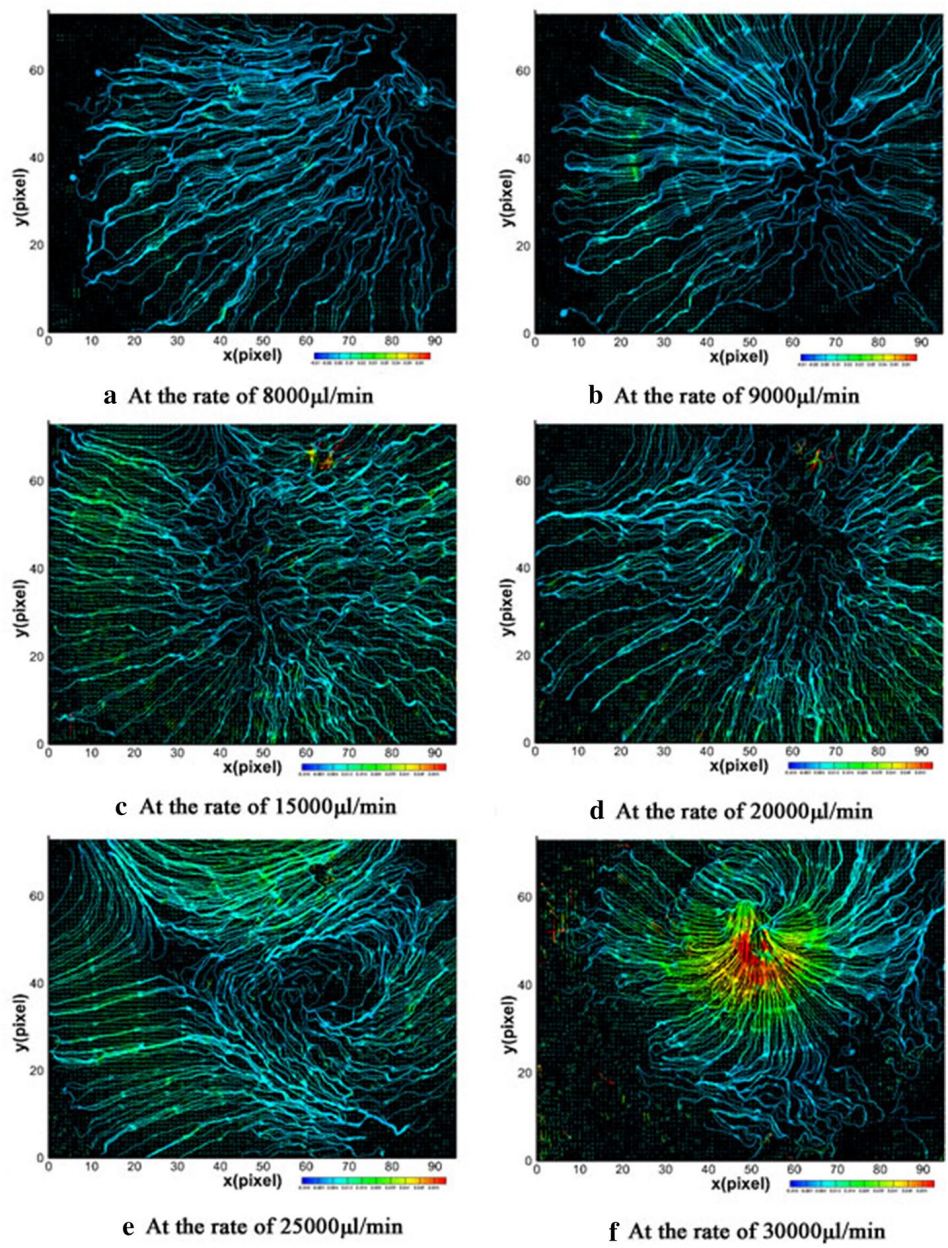
Velocity distribution measured at different speed

The distributions of flow fields in anterior chamber were gained in different inlet velocity. Figure 5 shows the results of PIV measurement in six different inlet velocities.

**Fig. 6:**
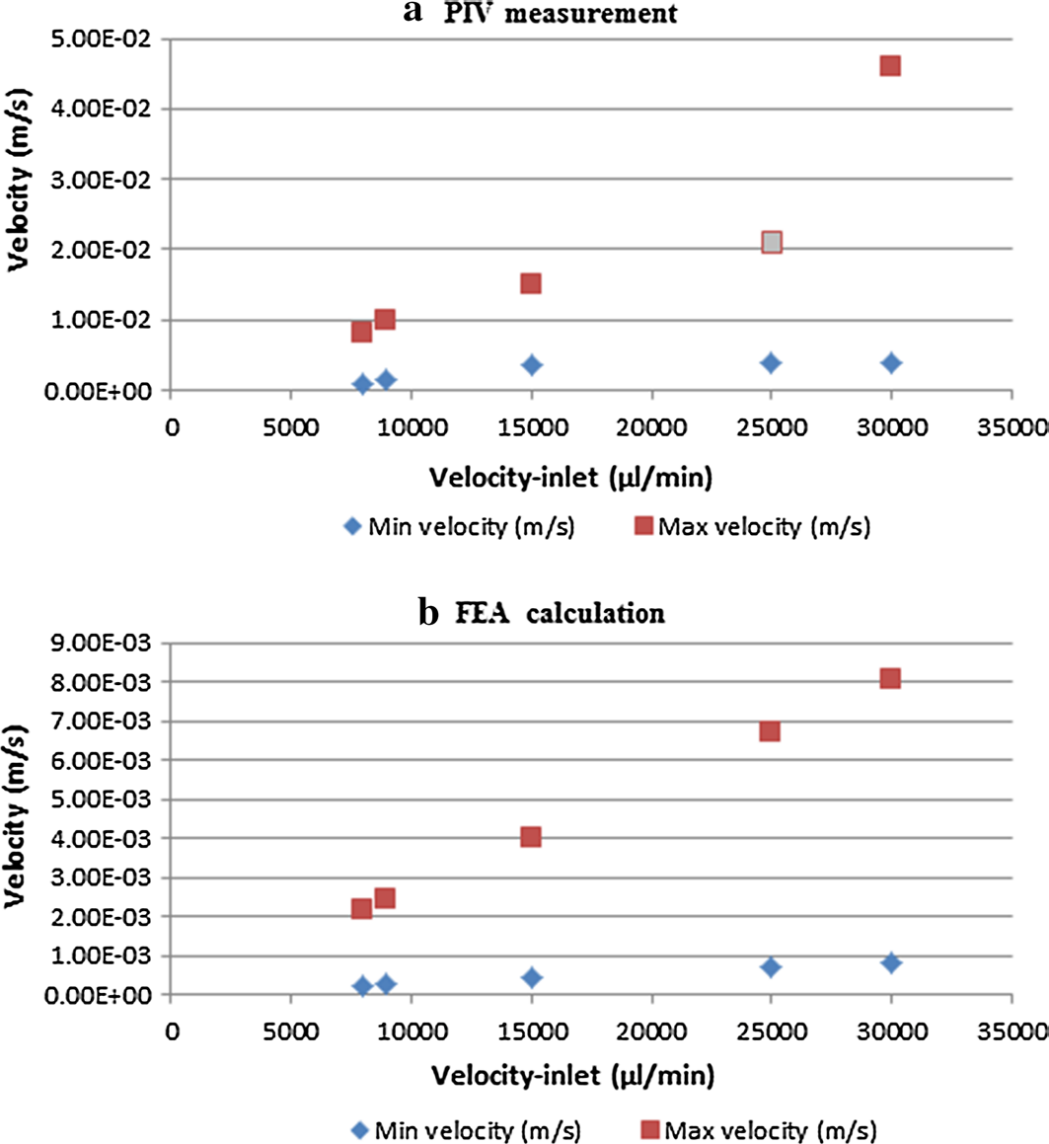
Scatter plots created by the data in Table 2

As shown in Figs. 5 and 6, the inlet velocity increased from 8000 to 30,000 µl/min. Flow fields are on the transverse plane at a distance of 10 mm from the XZ plane. From the distribution of vector in Fig. 5a, we can obtain the velocity values within 0.001–0.008 m/s, approximate to the values (0.000218–0.00215 m/s) calculated from ANSYS14.0 in the same initial conditions. The streamline on the interest plan is similar to the result from the finite element calculation, which is symmetric and divergence uniformed from the center of pupil to surrounding. Comparing the data in Table 2a, b under the same conditions, the maximum and minimum flow rates are in the same order of magnitude, and the distribution of streamlines is also approximately consistent. Ignored the value measurement of PIV at the rate of 25,000 µl/min, a disordered flow field shown in Fig. 5e, caused by the leakage of the experimental device. Thus, the effectiveness of the finite element analysis performed was verified by the comparison between the finite element analysis results and the test results.

**Table 2:** Velocity values on the transverse plan at a distance of 10 mm from the XZ plane in the supine position of anterior chamber

Velocity-inlet (µl/min)	(a) PIV measurement		(b) FEA calculation	
	Min velocity (m/s)	Max velocity (m/s)	Min velocity (m/s)	Max velocity (m/s)
8000	1.00E−03	8.00E−03	2.18E−04	2.15E−03
9000	1.50E−03	1.00E−02	2.46E−04	2.42E−03
15,000	3.50E−03	1.50E−02	4.09E−04	4.03E−03
25,000	3.80E−03	2.10E−02	6.80E−04	6.70E−03
3000	3.90E−03	4.60E−02	8.15E−04	8.04E−03

The values in (a) are measured by PIV experiment, and the corresponding values in (b) are obtained from FEA calculations

### Flows induced by high and normal IOP

The increase of the intraocular pressure could affect the aqueous humor fluid flow in anterior chamber. The working IOP conditions are 13.5 and 27 mmHg in our study to simulate the healthy model and the glaucomatous case, respectively. The values of aqueous humor fluid flow with different intraocular pressure are shown in Table 3.

**Table 3:** Values of aqueous humor obtained by the simulation of high and normal IOP with the gravity in horizontal and vertical directions

IOP(mmHg)	Maximum velocity (m/s)	Average velocity (m/s)	Pressure (Pa)	Temperature (°C)
In the horizontal position				
27	9.586e−4	3.89514e−5	3600–3611	34–37
13.5	9.439e−4	4.10097e−5	1800–1811	34–37
In the vertical direction				
27	9.941e−4	2.49185e−4	3600–3611	34–37
13.5	9.601e−4	2.50185e−4	1800–1812	34–37

**Fig. 7:**
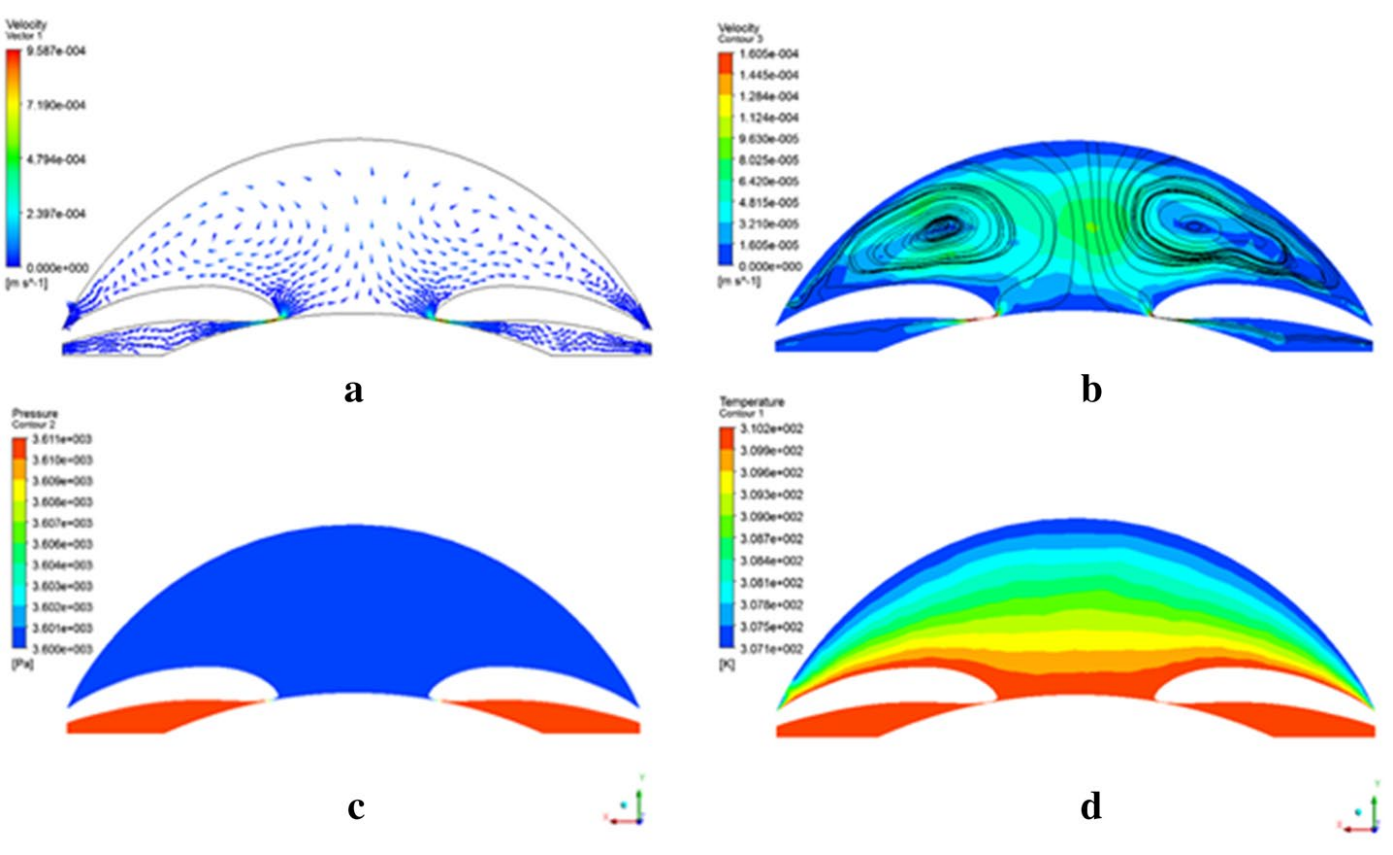
The velocity vector (**a**), streamline (**b**), pressure (**c**), and temperature (**d**) distribution with the gravity in the −y direction and the outlet pressure in 27 mmHg, which simulating the high IOP patient in supine position

In Fig. 7, the velocity, pressure, and temperature distributions are perpendicular to iris plan, with the gravity in the −y direction. This would be the case of a high IOP (27 mmHg) patient laying in the supine. The velocity of aqueous humor from ciliary body to the iris-lens gap increases up to 9.586e−4 m/s. After reaching anterior chamber, aqueous humor climbs towards the posterior surface of cornea, where the two counter-rotating vortices can be seen on the symmetry plane of the anterior chamber. The velocity increases up to 9.586e−4 m/s in the iris-lens gap, reached the maximum speed in anterior chamber. It is almost the lowest rate of flow close to cornea posterior surface. The average velocity in anterior chamber is up to 3.89514e−5 m/s. The pressure in posterior chamber is 3611 Pa, which is higher than value of 3600 Pa in anterior chamber. The differential temperature is approximately 3 °C between the cornea and lens. And the temperature counters would be symmetric about the radius. The distributions of velocity, pressure, and temperature in normal IOP model are similar to these of high IOP.

**Fig. 8:**
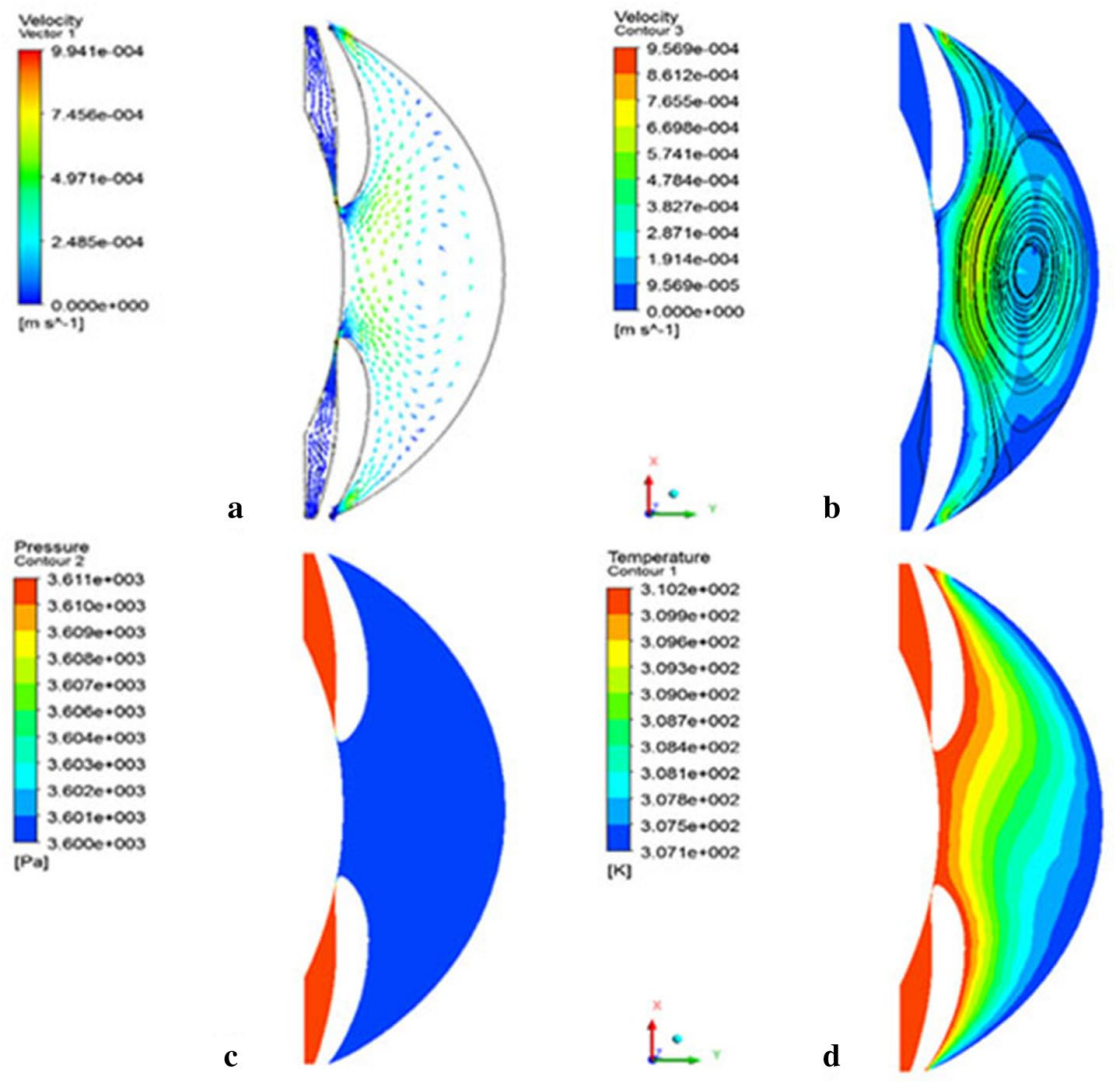
The velocity vector (**a**), streamline (**b**), pressure (**c**), and temperature (**d**) distribution in −x gravity direction and the outlet pressure in 27 mmHg, which simulating the high IOP patient in sitting position

Figure 8 shows the velocity, pressure, and temperature distributions in −x direction, simulating the sitting position of patient. The warmer aqueous humor rises upward along the iris surface to the iridocorneal angle. There aqueous humor could leave trabecular meshwork or move alone the posterior surface of cornea downward to the iridocorneal angle. Again there aqueous humor can leave via trabecular meshwork or arise along the iris surface. It gets re-mixed with aqueous humor formed a large vortex in the center of anterior chamber. Aqueous humor finally streams out of the trabecular meshwork. High velocities values are presented in the iris-lens gap (9.941e−4 m/s) and approach to the central portion of the anterior chamber (6.698e−4 m/s). The pressure in posterior chamber is higher than anterior chamber, and the pressure difference is about 11 Pa. The temperature of aqueous humor closed to posterior surface of cornea is about 34 °C, increased uniformity to the temperature of iris surface.

Distributions of velocities in the anterior chamber are similar in healthy and glaucomatous simulation calculations.

### Distributions on cornea and iris

**Fig. 9:**
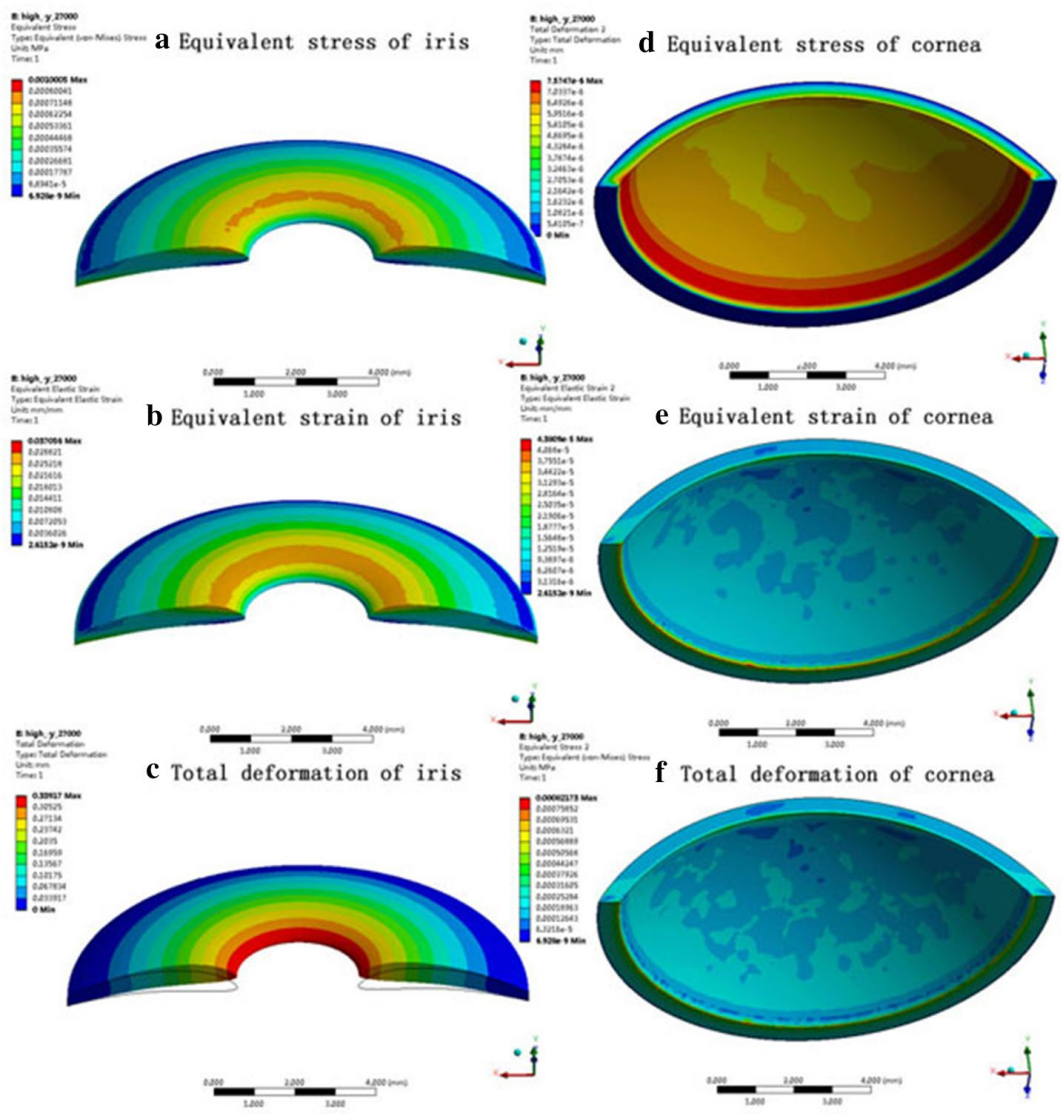
Distributions of iris and cornea interacted in the fluid flow with the gravity in −y direction and the outlet pressure in 27 mmHg

The values of equivalent stress, equivalent strain, and the total deformation of cornea and iris are represented in Table 4a, b. With the gravity directed along the −Y axis, the distributions on iris and cornea in high IOP model are shown on Fig. 9. The Magnitude of equivalent tress was found to be higher at the thicker part of the anterior surface of the iris along a horizontal band, whereas the peak arrived at the mid of cornea. In this case, equivalent tress is not influenced by the elasticity modules of iris. On the other hand, the equivalent stress contours of the iris (Fig. 9a) and cornea (Fig. 9d) come to a minimal difference between the vertical and the horizontal position of the eye. The maximum equivalent stress in the vertical orientation is slightly smaller than it is in the horizontal position.

**Table 4:** Values of iris obtained by the simulation of high and normal IOP with the gravity in −Y and −X directions

Iris elasticity modulus (Pa)	Equivalent stress (MPa)	Equivalent strain	Total deformations (mm)
(a) In the horizontal position			
IOP = 27 mmHg			
27,000	1.0005e−3	0.037056	0.33917
32,400	1.0005e−3	0.03088	0.28264
54,000	1.0005e−3	0.018528	0.16959
IOP = 13.5 mmHg			
27,000	1.4602e−3	0.035289	0.3597
32,400	1.4602e−3	0.029407	0.29975
54,000	1.4602e−3	0.017644	0.17985
(b) In the vertical direction			
IOP = 27 mmHg			
27,000	9.999e−4	0.037033	0.33869
32,400	9.999e−4	0.030861	0.28224
54,000	9.999e−4	0.018517	0.169853
IOP = 13.5 mmHg			
27,000	9.7102e−4	0.035964	0.3671
32,400	9.7102e−4	0.02997	0.30591
54,000	9.7102e−4	0.017982	0.18555

We also show the corresponding equivalent strain values in Table 4, and this reveals significant changes due to iris elasticity modulus. The maximum equivalent strain is at the middle of cornea. However, while the equivalent strain of iris achieved the peak at the thicker part of the anterior surface of the iris. Compared the results of various situations, we found that the equivalent strain was higher in horizontal orientation than that of in vertical position. With the increase of elasticity modulus of iris, the equivalent strain of iris decreased at almost the same rate.

Through the analysis of fluid–structure interaction, we can also see the presence of IOP makes some difference to the total deformation of iris. The maximum deformation with a value of 0.33917 mm (Fig. 9c) is in peripheral iridotomy, whereas the peak is reached to the iridocorneal angle of cornea in horizontal orientation. We also note that the deformation of iris varies from the changes of the iris elasticity modulus. However, the deformation of the iris in high IOP is lower than that of in normal.

The corresponding equivalent strain with the value of 4.3809e−5 for cornea (Fig. 9e) is too small, while the deformation of cornea (Fig. 9f) is about 7.5747e−6 mm, all of which can be ignored.

## Discussion

**Fig. 10:**
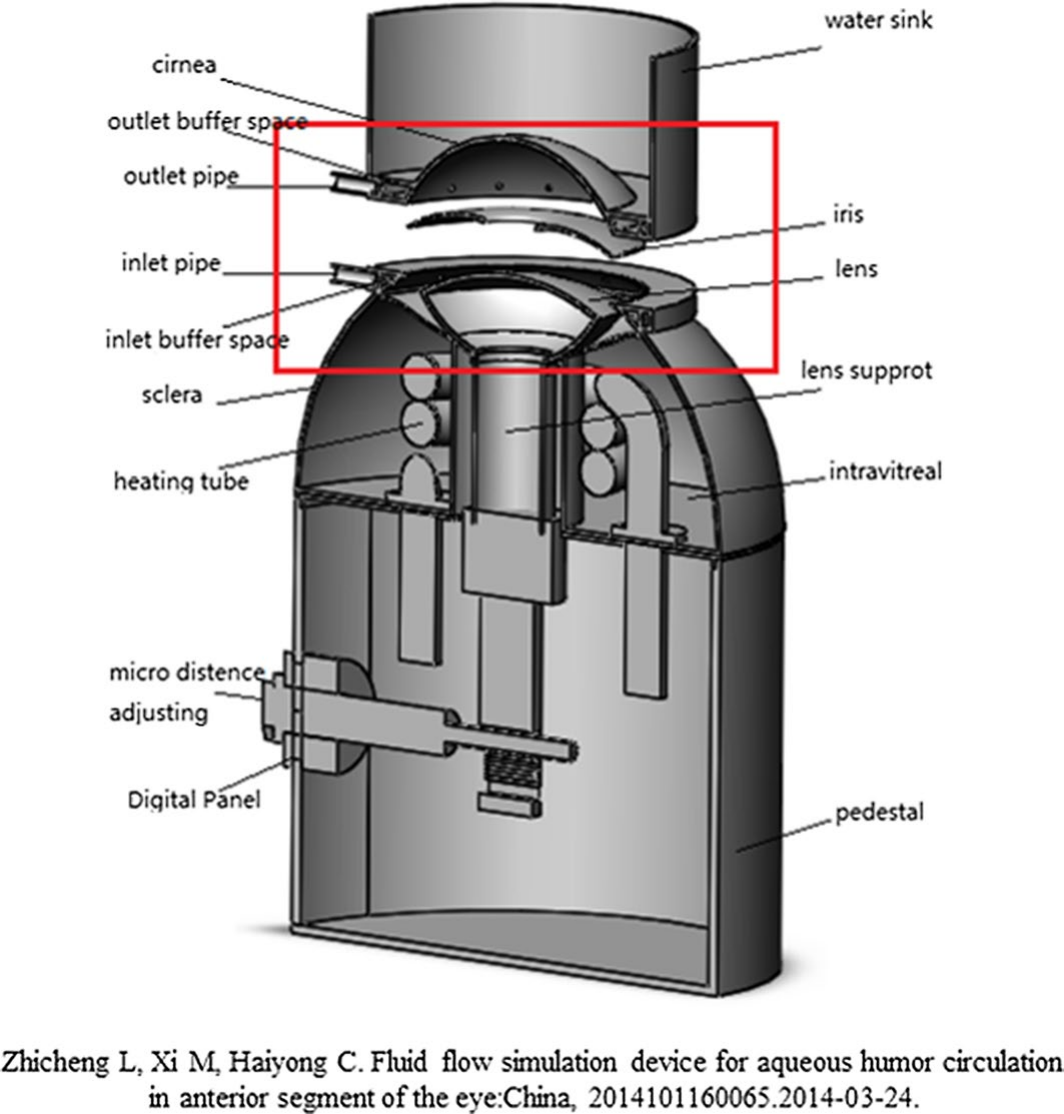
A 3D model built with a heating tube and a macro distance adjusting part, based on the model in Fig. 3a

In this paper, the verification of calculation has been performed by PIV experiments. In addition, the simulation device can simulate the flow of aqueous humor visually and effectively. To achieve this goal, we built a Three D model (Fig. 10) added with a heating tube and a macro distance adjusting part, hoping to simulate the thermal in human eye and the morphology characteristics of pupil block in further research.

To explain the similarity of the flow in PIV experiment, a model can be treated similitude with the real object if the two share similarities of geometric, kinematic and dynamic [23]. It can be regarded similar if the two applications both have the same geometrical shape or one has the particular uniform scaling of the other. In our study, the model and real human eye are geometrically similar based on their corresponding linear proportionate dimension scales. Moreover, flows in the two systems are Kinematically similar when the velocity ratios *v*_*m*_ and *v* are the same for all pairs of corresponding, or homologous, points. In addition, dynamically similar is defined based on both geometric similarity and kinematic similarity. An important conclusion of fluid mechanics is that incompressible, isothermal flows in or around the similar geometric bodies are considered dynamically similar if the two have the same Reynolds number *Re*, where *Re* represents the ratio of inertial to viscous forces. In order to prove the similarity of two flows, the Reynolds numbers and Euler numbers must have been equal. When comparing fluid behavior at corresponding points in a model and a full-scale flow, the following holds:

4 $$Re_{m} = Re\quad {\text{i}}.{\text{e}}. \frac{{\rho_{m} r_{m} v_{m} }}{{\eta_{m} }} = \frac{\rho rv}{\eta }$$

5 $$Eu_{m} = Eu\quad {\text{i}}.{\text{e}}. \frac{{P_{m} }}{{\rho_{m} v_{m}^{2} }} = \frac{P}{{\rho v^{2} }} ,$$

where *ρ* stands for fluid density, *r* defines body radius, *v* is stream velocity, *η* is fluid viscosity, and *P* is the local pressure. The flow around the model is represented by m, and the other is the actual flow.

We use normal saline to simulate the Physiological aqueous humor fluid in model experiment, in order ensure the same fluid mechanics properties in model and prototype [24]. That is to say model m and the actual flow have the same fluid density and stream viscosity. In particular fluid dynamics, the volumetric flow rate is the volume of fluid which passes per unit time, usually represented by the symbol *Q*. Volumetric flow rate can be defined by:

6 $$Q = \pi r^{2} v,$$

where *r* is body radius, *v* is stream velocity. The model is magnified in 5 times in radius scales in PIV experiment. Thus by combining Eqs. (4) and (6), the resulting equation is:

7 $$Q_{m} = 5 Q.$$

Due to the low rate of aqueous humor fluid flow, the difference in *v*_*m*_ and *v* could be neglect in Eq. (5). The similarity of the flow in model and human eye could be proved, when using the inlet volumetric flow rate *Q*_*m*_ (15 µl/min) in PIV experiment. And we will achieve this low speed measurement in the following experiments.

The study on the visualization of the flow field distribution, the experimental model will be amplified to a certain degree based on the Reynolds number similarity principle. Because the human eye is small, and the fluid flow rate of aqueous humor is relatively low, there is very little experimental method to verify the validity of the model. 5 times amplified model is more easily carried out PIV experiment for the rate of infusing is larger than the secreted rate of aqueous humor in real eye, while we are trying to validate under a real scale model. To verify, we focus on the fluid streamlines, and the flow field distribution. The measured fluid distributions are similar to the results obtained by finite element method, so it can be explained that this method is valuable, and it is feasible to continue to improve in the verification experiment. We plan to improve the simulation model and use the advanced experimental equipment to complete the measurement of the flow field in low speeds.

The values of velocity and pressure of aqueous humor formulated in steady state makes no difference in horizontal position or vertical orientation. Therefore, under the rapid eye movement, the study of the mixing aqueous humor in the anterior chamber becomes more meaningful. Modarreszadeh [25] carried out a detailed simulation study on this issue. The importance of the driving mechanism for aqueous flows was considered in our study. We find that environmental temperature could increase the maximum velocity of aqueous humor, while produce no significant increase in pressure difference.

**Fig. 11:**
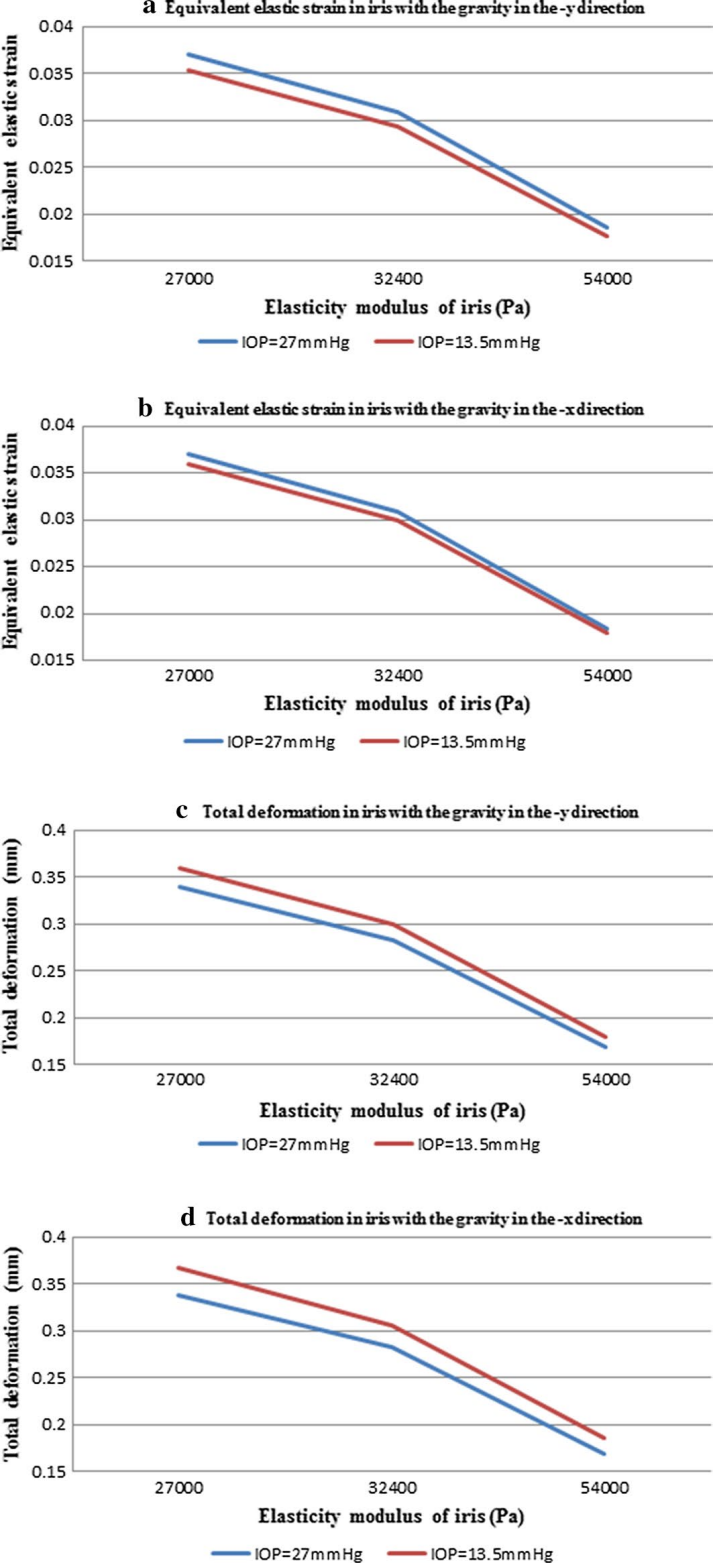
Line charts created by the data in Table 4

The relationship between the deformation of iris and the intraocular pressure is of the most interest in this study. Ritch [26] argued that the underlying mechanism behind PACG is pupillary block. In that case, a flow resistance is generated from the narrow gap between iris and lens. The resulting pressure drop between anterior chamber and posterior chamber forces the iris bomb, significantly narrowing the ACA and impeding aqueous humor outflow. During the research, Mapstone [27] proposed that a “pupil-blocking force” is raised in pupillary block, and the amount of posterior forces exerted by material stretch and iris muscles, keeping the iris tip attach the lens surface despite a counteracting pressure difference. Mapstone suggested that, pupil-blocking force will increase if the lens positioned more anteriorly, thickness growth or curvature change. This assertion is supported by the clinical observation that an intumescent lens can induce pupillary block. In our simulation shown in Fig. 11, equivalent strain in iris is decreased with the increase in the elastic modulus of iris. Under the same conditions, the value of equivalent strain in high IOP is higher than that of in low IOP. The total deformation of iris induced in the high IOP model due to the rise of elastic modulus of iris was lower than that of the normal IOP. The composition of the iris with the gravity in the −x direction is similar with the −y direction. That is to say, with high intraocular pressure, the displacement of the iris pupillary area is lower, while the ability of the iris deformation is higher than that of in low intraocular pressure. Therefore, the risk of pupillary block (the distance between iris and cornea is higher), iris bombe, and angle closure (the iris arched itself leads to a shallow angle) is increased in high IOP. This could be the explanation of the relationship between the high intraocular pressure and deformation of iris. The elevated intraocular pressure increased the trend of the iris deformation, which affect the normal aqueous humor outflow.

## Conclusion

Our study provides a means to verify the validity and reliability of the finite-element analysis model. Through PIV experiment, we have captured the flow field in anterior chamber in the simulation device and calculated the corresponding velocity distributions. The valid model of idealization geometry of human eye can be helpful to investigate the relationship among localization, the changes of iris elasticity modulus and high IOP, so as to make further exploration in the pathogenesis of glaucoma.
