# Erratum to: Fluid and structure coupling analysis of the interaction between aqueous humor and iris

**DOI:** 10.1186/s12938-017-0312-4

**Published:** 2017-01-19

**Authors:** Wenjia Wang, Xiuqing Qian, Hongfang Song, Mindi Zhang, Zhicheng Liu

**Affiliations:** 1 School of Biomedical Engineering, Capital Medical University, Beijing, China; 2 Beijing Key Laboratory of Fundamental Research on Biomechanics in Clinical Application, Capital Medical University, Beijing, China; 3 School of Mechanical and Vehicular Engineering, Beijing Institute of Technology, Beijing, China

## Erratum to: BioMed Eng OnLine 2016, 15(Suppl 2):133 DOI 10.1186/s12938-016-0261-3

Upon publication of the article [1], it was noticed that the Funding information, in the Declarations section, was incorrect. The correct information is now provided below in this erratum.
